# Molecular subgroups of human malignant peripheral nerve sheath tumors are conserved in canines

**DOI:** 10.1016/j.neo.2026.101368

**Published:** 2026-09-13

**Authors:** Houria Ech-Cherif, Sharareh Bancroft, Daniel Fuchs, Lennart Opitz, Armin Jarosch, Marie-Therèse König, Luca Aresu, Greta Foiani, Anne Flörcken, Kyle Williams, David A. Largaespada, Franco Guscetti, Mirja C. Nolff, Enni Markkanen

**Affiliations:** a Institute of Veterinary Pharmacology and Toxicology, Vetsuisse Faculty, University of Zurich, 8057, Zurich, Switzerland; b Functional Genomics Center Zürich, ETH Zurich/University of Zurich, 8057, Zurich, Switzerland; c Institute of Pathology, Charité-Universitätsmedizin Berlin, 10117, Berlin, Germany; d Institute of Veterinary Pathology, Vetsuisse Faculty, University of Zurich, 8057, Zurich, Switzerland; e Department of Veterinary Sciences, University of Turin, Grugliasco, Italy; f Laboratory of Histopathology, Istituto Zooprofilattico Sperimentale delle Venezie, 35020, Legnaro (Padua), Italy; g Department of Hematology, Oncology, and Tumor Immunology, Charité-Universitätsmedizin Berlin, 13353, Berlin, Germany; h Department of Pediatrics, Masonic Cancer Center, University of Minnesota, Minneapolis, MN, USA; i Small Animal Surgery Tierspital Zürich, Vetsuisse Faculty, University of Zurich, 8057, Zurich, Switzerland

**Keywords:** Comparative oncology, Transcriptomics, Canine cancer model, Oncogenic signaling pathways, Malignant peripheral nerve sheath tumors, Dog tumor

## Abstract

Malignant peripheral nerve sheath tumors (MPNST) are aggressive sarcomas of Schwann cell lineage with poor prognosis in both humans and dogs. While rare in humans, MPNSTs occur more frequently in dogs and share histomorphological and clinical features. Recent methylome and transcriptome analyses have identified two molecular subgroups of human MPNST with distinct oncogenic signaling pathways and prognostic implications; however, it remains unclear if these subgroups also exist in canines. Given their higher incidence and biological similarities to human disease, canine MPNSTs represent a promising comparative model to investigate molecular subtypes and evaluate novel therapeutic strategies. To characterize canine MPNST and assess molecular parallels with the human subgroups, we applied laser-capture microdissection (LCM) followed by RNAsequencing to analyze tumor tissue from 20 canine MPNST. Principle component and differential gene expression analyses identified two clearly distinct transcriptional clusters corresponding to spindle cell and epithelioid MPNST variants, respectively. Unsupervised cross-species comparison aligned the two canine clusters with the human G1 and G2 subgroups. Accordingly, one cluster was characterized by SHH pathway activation and increased cell cycle activity, while the other showed non-canonical WNT pathway, Schwann cell–like features and marked macrophage infiltration. Immunohistochemistry further demonstrated loss of H3K27me3, p-ERK activation and β-catenin signaling by IHC in a subset of tumors. These findings support the value of canine MPNST as clinically amenable model for structured assessment of novel therapeutic approaches to benefit patients of both species.

## Highlights

• Largest RNAseq dataset on canine MPNST enables the first discovery of molecular subtypes in canine MPNST.
• Molecular subtypes of human MPNST are conserved in canines.
• Canine MPNST represent faithful immunocompetent models for preclinical therapeutic evaluation of novel therapies.

## Introduction

Malignant peripheral nerve sheath tumors (MPNST) represent aggressive soft-tissue sarcomas of Schwann cell lineage affecting both humans and dogs alike. While rare in humans, MPNSTs are moderately frequent in dogs [1,2]. Approximately 50% of human MPNST are associated with the cancer predisposition syndrome Neurofibromatosis Type 1 (*NF1*) [3]; it remains unclear whether a comparable *NF1* syndrome exists in canines. Long-term prognosis for MPNST in both species is unfavorable, with reported 5-year overall survival rates for humans ranging from 30% to 60% [4]. The rarity of human MPNST, combined with biological heterogeneity and a lack of effective targeted therapies, poses significant diagnostic and therapeutic challenges, underscoring the need for improved molecular stratification and novel therapies. Recent methylome and transcriptome profiling studies have identified two distinct molecular subgroups of human MPNST (G1 and G2) that differ in targetable oncogenic pathways and prognosis [5]. MPNST-G1 are characterized by activation of SHH pathway, while MPNST-G2 show active WNT/ß-catenin/CCND1 signaling.

A significant obstacle in understanding MPNST biology and evaluating novel therapeutic strategies is the lack of reliable and relevant models for structured clinical assessment. Here, canine MPNST represent an interesting possibility, given their higher incidence, shared histomorphological and clinical features with human MPNST, and the fact that canine tumors offer a unique opportunity to assess novel anti-cancer treatments in the context of naturally occurring drug resistance, metastasis and tumor-host immune interactions that are difficult to recapitulate otherwise. We therefore set out to assess whether human MPNST have a molecular correlate in canine MPNST.

## Material and methods

### Ethics approval and consent to participate

No animals were killed for the purpose of this research project, as the tissue analyzed had been surgically removed in a curative setting with the consent of the patient owners. According to the Swiss Animal Welfare Law Art. 3 c, Abs. 4 the preparation of tissues in the context of agricultural production, diagnostic or curative operations on the animal or for determining the health status of animal populations is not considered an animal experiment and, thus, does not require an animal experimentation license. The FFPE material from canine patients was obtained for diagnostic reasons and its use for research therefore does not require ethical approval in Switzerland in accordance with the national and local legislation and institutional requirements.

### Selection of canine cases for LCM

FFPE archival tissue samples of n = 20 canine MPNST were retrieved from the pathology archives of the Institute of Veterinary Pathology of the Vetsuisse-Faculty of the University of Zurich, the Department of Veterinary Sciences, University of Turin and the Laboratory of Histopathology of the Istituto Zooprofilattico Sperimentale delle Venezie. Cases comprised biopsies or surgical resections with the diagnosis of canine MPNST that were selected by certified pathologists (FG, LA, GF) according to histomorphological criteria [14] and expression of at least one of the markers GFAP, SOX10 or NGFR. Paraffin blocks were routinely kept at room temperature. All cases and diagnoses were reviewed by a veterinary pathologist (FG). Supplementary Table 1 provides clinical details, such as age and breed of each patient, sample age and tumor type, for all cases included in the study.

### Laser-capture microdissection (LCM), RNA isolation and quality control

Tissue processing for LCM was performed as originally described [15]. In short, sections of 10 μm thickness mounted on PEN membrane glass slides (Thermo Scientific) were used to isolate areas of interest using the ArcturusCellect^TM^ Laser Capture Microdissection System (Thermo Scientific) according to the manufacturer’s protocol. Areas to be isolated were identified by a specialist pathologist on a consecutively cut 3 μm section that was stained with Hematoxylin-Eosin to support detailed histomorphologic assessment of the tissue at hand. Isolation of areas and tissue of interest was verified by microscopic examination of the LCM cap as well as the excised region after microdissection. Two caps were collected per case for RNA isolation. After excision, sterile blades and forceps were used to peel off the thermoplastic membranes containing captured tissue from the cap, which were then transferred into sterile Eppendorf® Safe-Lock tubes and frozen at −20°C until further processing. Subsequently, RNA was isolated using the Covaris® truXTRAC FFPE RNA kit and the Covaris® E220 focused ultrasonicator as established [15]. RNA abundance and quality was analyzed using the 4200 Station Software using the High Sensitivity RNA ScreenTape kit (Agilent Technologies), according to the manufacturer’s protocol.

### RNA sequencing

RNA library preparation and ribosomal RNA depletion was performed using 10 ng of RNA and the SMARTer Stranded Total RNAseq Kit V2-Pico Input Mammalian (Clontech/Takara Bio USA) according to the manufacturer’s protocol. Paired-end-read sequencing (150 bp) was performed in a single batch for all samples using the Illumina NovaSeq X Plus according to standard protocols of the Functional Genomics Center Zurich (FGCZ). RNAseq data processing and analysis was performed as detailed in Supplementary Materials and Methods.

## Results

**Fig. 1:**
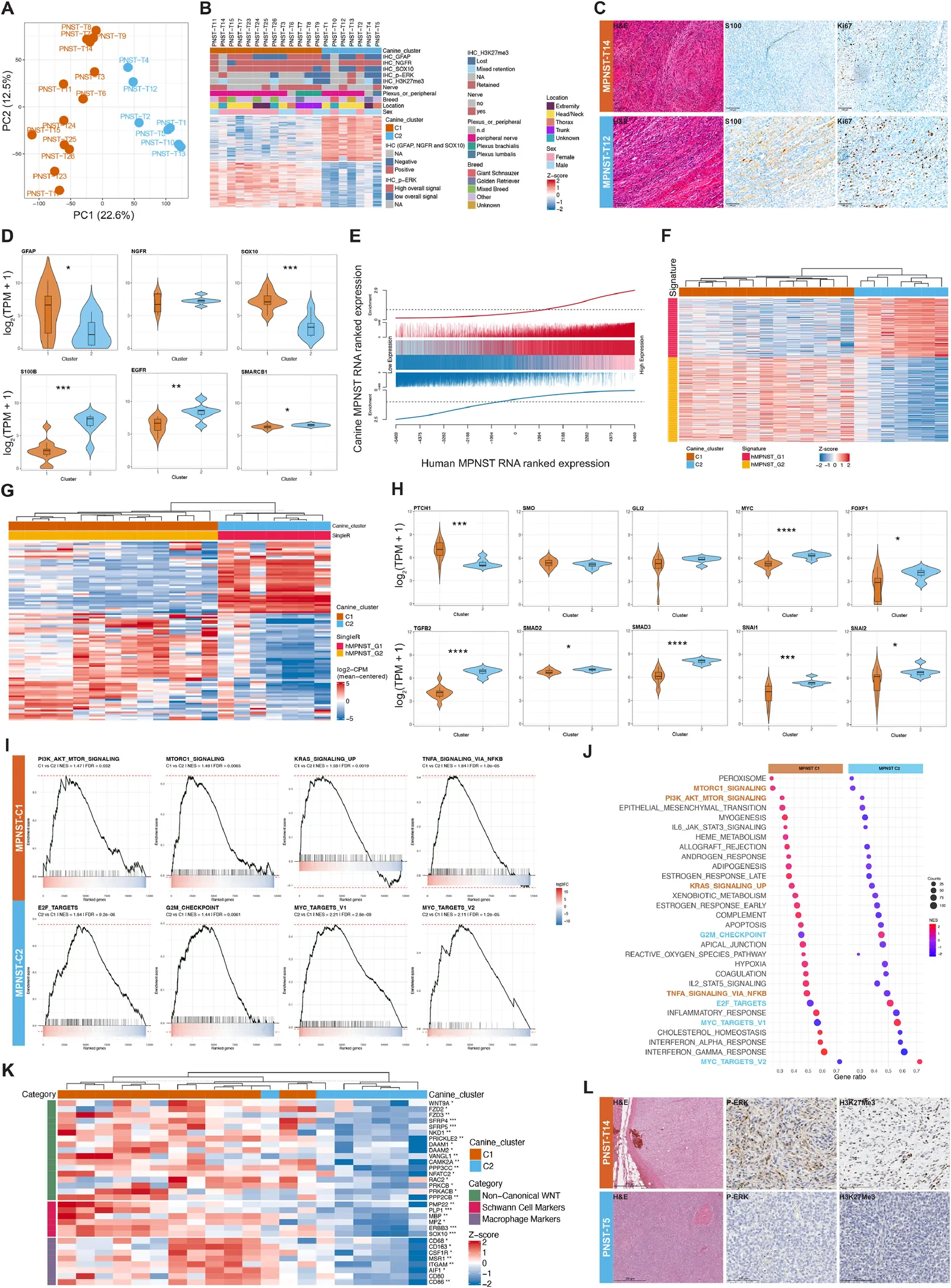
**Molecular characterization of canine malignant peripheral nerve sheath tumors (MPNST) identifies two transcriptionally distinct subtypes with human MPNST correlates.** (A) Principal component analysis (PCA) of canine MPNSTs using all transcripts. (B) Heatmap of the top 500 upregulated genes from each cluster. Top annotation includes clinical information and IHC results. (C) Representative IHC images of canine MPNST subtypes: spindle cell variant (MPNST-T14, C1) and epithelioid variant (MPNST-T12, C2). (D) Transcript expression of select MPNST markers in C1 vs C2. Welch's two-sample t-test; Asterisks denote significance based on adjusted P values: *adj. P < 0.05, **adj. P < 0.01, ***adj. P < 0.001. (E) Competitive gene set testing to compare canine and human MPNST expression profiles. (F) Unsupervised hierarchical clustering of canine MPNST samples using a human MPNST G1/G2 differential expression signature. (G) SingleR annotation to assign canine tumors to human MPNST subgroups G1 and G2, respectively. (H) Transcript levels in C1 vs C2 of Sonic Hedgehog, TGFβ, and EMT-associated signaling pathways. Welch’s two-sample t-test; Asterisks denote significance based on adjusted P values: *adj. P < 0.05, **adj. P < 0.01, ***adj. P < 0.001 and ****adj. P < 0.0001. (I) GSEA enrichment plots of PI3K–AKT–mTOR, KRAS, TNFα signaling via NF-κB in C1 vs C2 (top), and E2F targets, G2M checkpoint, and MYC targets (v1 and v2) signaling in C2 vs C1 (bottom). (J) GSEA plots for top subtype-specific enriched pathways in C1 vs C2. (K) Unsupervised heatmap of transcripts associated with non-canonical WNT pathway, Schwann cell markers, and macrophages in all samples. (L) Representative IHC images of H3K27me3 and p-ERK staining in canine MPNSTs.

We applied laser-capture microdissection (LCM) followed by RNAsequencing [6] to specifically analyze tumor tissue from archival FFPE samples of 20 histopathologically confirmed cases of canine MPNST across 13 different dog breeds (Supplementary Table 1). Principle component analysis (PCA) identified 2 clearly distinct transcriptional clusters (Fig. 1A, hereafter referred to as cluster C1 and C2, respectively), reminiscent of the transcriptomic clustering of human MPNST into G1 and G2. The two groups were clearly differentiated based on gene expression (Fig. 1B). Independent assessment of a case from each cluster (T-14 for C1, T-12 for C2) by a physician pathologist (AJ) diagnosed T-14 as MPNST spindle cell variant and T-12 as MPNST epithelioid variant (Fig. 1C). Though cases were IHC-positive for SOX10, NGFR and/or GFAP (Fig. 1B), mRNA levels of SOX10 and GFAP was significantly higher in C1 compared to C2, consistent with more Schwann cell-like characteristics in C1 (Fig. 1D). In contrast, C2 showed significantly higher expression of S100B, SMARCB1 and EGFR, suggesting more precursor-like cell characteristics (Fig. 1D) [7,8].

We next assessed interspecies similarity between canine and human MPNST using a transcriptomic dataset of 12 human MPNST [5]. Comparative gene set enrichment analysis revealed that the 50% highly expressed genes in canine MPNST (red vertical bars) were strongly enriched towards highly expressed genes in humans (right side) and vice versa for the lowly expressed transcripts, demonstrating a very high correlation and wide-ranging conservation in gene expression between the two species (Fig. 1E). To compare the canine clusters with the human subgroups, we derived a signature of differentially expressed genes in G1/G2 that were also detected in the canine dataset and applied it to perform unsupervised hierarchical clustering of canine samples (Fig. 1F). Strikingly, this clearly separated the samples into the two canine clusters, with C1 corresponding to human G2, and C2 to human G1, respectively. This was further corroborated by interspecies comparison using SingleR (Fig. 1G) [9]. As in human G1, canine C2 tumors were characterized by a strong activation of SHH signaling, through a decrease in expression of the inhibitory factor PTCH1, which - via SMO and GLI2 - results in a significantly elevated expression of transcription factors MYC and FOXF1, further reinforced by an increase in TGFB2-SMAD2/3 that culminates in upregulation of EMT-associated factors SNAI1/2 (Fig. 1H). This functionally results in increased cell cycle activity in C2 tumors, as evidenced by significant enrichment of Hallmark_E2F_Targets and G2M_Checkpoint in C2 over C1 (Fig. 1I, J). In agreement with changes specific to human G2, canine C1 highly expressed components of the non-canonical WNT pathway, including WNT9A, RAC2, FZD2/3, and CAMK2A (Fig. 1K). C1 tumors further showed significant enrichment of TNFA signaling via NF-κB and PI3K–AKT–mTOR signaling (Fig. 1I, J), both of which have been implicated in enhancing non-canonical WNT signaling [10,11]. Consistent with human MPNST-G2 exhibiting Schwann cell–like features, C1 tumors demonstrated significant upregulation of Schwann cell markers PMSP2, PLP1, MBP, MPZ, ERBB3, and SOX10 (Fig. 1K). Furthermore, macrophage-associated markers CD68, CD163, CSF1R, MSR1, ITGAM, AIF1, CD80, CD86 were significantly upregulated, paralleling observations of increased macrophage infiltration in human G2 tumors [5].

A significant fraction of human MPNST displays loss of Polycomb repressive complex 2 (*PRC2*) caused by bi-allelic inactivation of *SUZ12* or *EED*, leading to loss of H3K27me3 [12]. IHC staining for H3K27me3 in a subset of 8 samples identified 3 cases in which staining was completely lost, while the other 5 showed a mixed retention of signal (Fig. 1L). Activation of the Ras–MAPK pathway with robust p‑ERK expression is common in human MPNST [13], but the presence of comparable signaling activity in canine MPNST has not been established. In our cohort, strong p‑ERK immunoreactivity was observed in 2 of 8 canine cases (Fig. 1L). Macrophage infiltration (based on Iba-1) was moderate to high in all samples, and β-catenin ranged from moderate to high, and p-SAPK-JNK stained weakly positive in two of the cases (Supplementary Table 1).

## Discussion

As such, this represents the largest transcriptomic dataset of histologically confirmed canine MPNST to date. Importantly, we found that canine MPNST can be classified in two molecular subgroups that are highly comparable to their human counterparts, and loss of H3K27me3, p-ERK activation, strong macrophage infiltration and β-catenin signaling can be detected in these tumors. These findings support the value of canine MPNST as clinically amenable model for structured assessment of novel therapeutic approaches for MPNST to benefit patients of both species.

## Originality statement

The data presented in this manuscript has not been previously published.

## Research funding

Work in the lab of E.M. is financially supported by the Swiss National Science Foundation (Grants no 10004833 and 10002567), the AKC Canine Health Foundation (CHF; grants No 03017 and 03228), the Albert-Heim-Stiftung (project No 157), and the Morris Animal Foundation (Grant no D24CA-046). Work in the lab of M.C.N. is supported by the Swiss National Science Foundation (Grant no 10002503), the AKC Canine Health Foundation (CHF; grant No 03019), the Gesellschaft zur Förderung von Kynologischer Forschung, the Albert-Heim-Stiftung (project No 146), the Forschungskredit der Universität Zürich, and the Bachofner legat. D.A.L. is supported by an American Cancer Society Research Professorship.

## Data availability

The RNA-seq data generated in this study is available in the Gene Expression Omnibus (GEO) under accession number GSE325901. The Human MPNST bulk RNA data (GSE207399) was downloaded from [https://www.ncbi.nlm.nih.gov/bioproject/PRJNA855245].

## CRediT authorship contribution statement

**Houria Ech-Cherif:** Formal analysis, Investigation, Methodology, Validation, Writing – original draft. **Sharareh Bancroft:** Formal analysis, Investigation, Methodology. **Daniel Fuchs:** Data curation, Formal analysis, Methodology, Visualization, Writing – review & editing. **Lennart Opitz:** Data curation, Formal analysis, Methodology, Software. **Armin Jarosch:** Formal analysis, Methodology, Validation, Writing – review & editing. **Marie-Therèse König:** Investigation, Methodology, Validation. **Luca Aresu:** Methodology, Resources, Writing – review & editing. **Greta Foiani:** Methodology, Resources, Writing – review & editing. **Anne Flörcken:** Formal analysis, Methodology, Validation, Writing – review & editing. **Kyle Williams:** Formal analysis, Investigation, Methodology, Validation, Writing – review & editing. **David A. Largaespada:** Formal analysis, Methodology, Validation, Writing – review & editing. **Franco Guscetti:** Formal analysis, Investigation, Methodology, Validation, Writing – review & editing. **Mirja C. Nolff:** Conceptualization, Formal analysis, Funding acquisition, Project administration, Resources, Supervision, Writing – review & editing. **Enni Markkanen:** Conceptualization, Formal analysis, Funding acquisition, Investigation, Project administration, Resources, Supervision, Writing – original draft, Writing – review & editing.

## Declaration of competing interest

Dr. Largaespada is the co-founder and co-owner of NeoClone Biotechnologies, Inc., Discovery Genomics, Inc. (recently acquired by Immunsoft, Inc.), and B-MoGen Biotechnologies, Inc. (recently acquired by the Biotechne corporation), and Luminary Therapeutics, Inc. He consults for Genentech, Inc., which is funding some of his research. The business of all these companies is unrelated to the contents of this manuscript. All other authors declare no competing interests.
