# Supplementary material for: Molecular subgroups of human malignant peripheral nerve sheath tumors are conserved in canines
Source: Neoplasia. 2026 Sep 13;81:101368. doi: 10.1016/j.neo.2026.101368 (PMC13587806; doi:10.1016/j.neo.2026.101368)
Supplement: Supplementary file 1 [file mmc1.pdf]

# Molecular Subgroups of Human Malignant Peripheral Nerve Sheath Tumors are Conserved in Canines

Houria Ech-Cherif[1], Sharareh Bancroft[1], Daniel Fuchs[1], Lennart Opitz[2], Armin Jarosch[3], Marie-Therèse König[4], Luca Aresu[5], Greta Foiani[6], Anne Flörcken[7], Kyle Williams[8], David A. Largaespada[8], Franco Guscetti[4], Mirja C. Nolff[9,#] and Enni Markkanen[1,#]

## Supplementary Materials and Methods

### RNAseq data processing

The raw reads were cleaned by removing adapter sequences, trimming low-quality ends, and filtering reads with low quality (phred quality < 20) using Trimmomatic (version 0.36) [1]. Sequence pseudoalignment of the resulting high-quality reads to the canine reference genome (Canis_familiaris/Ensembl/ROS_Cfam_1.0/Annotation/Release113-2025-07-02) and quantification of gene-level expression was carried out using Kallisto (version 0.44) [2]. Gene counts were imported into the R/Bioconductor package EdgeR [3] (R, version 3.6.1, EdgeR, version 3.28), and trimmed mean of M values normalization size factors were calculated to adjust for samples for differences in library size. The generalized linear model was used to detect differentially expressed genes incorporating adjusted (Benjamini and Hochberg method) P-values using the limma package in R [4].

### Principal component analysis

Principal component analysis was performed using the prcomp function in R on log2-transformed gene expression values from tumor samples. Visualization of principal components was carried out in R using the ggplot2 package [23], with PC1 and PC2 represented on the x- and y-axes, respectively.

### Heatmap

Differential gene expression analysis between transcriptomic clusters (C1 vs C2) was performed based on genes filtered for an adjusted p-value (Benjamini–Hochberg FDR < 0.05) and |log2 fold change| > 1, and the top 500 differentially expressed genes were included in the signature. Gene expression values were z-score scaled across samples, and the heatmap was generated using the ComplexHeatmap package [24,25] with hierarchical clustering based on Euclidean distance and column annotations indicating transcriptomic clusters and clinicopathological variables.

### Violin plots

Violin plots were generated in R using the ggplot2 package [26] to visualize log2-transformed expression values (log2[TPM + 1]) across transcriptomic clusters. Distributions were displayed as violin plots with overlaid boxplots. Statistical comparisons between groups were performed using Welch's two-sample t-test, and p-values were calculated and annotated using the ggpubr package.

### Barcodeplot

Cross-species comparative analysis of tumor-specific expression was performed using the barcode enrichment plot from limma [22] and an external dataset of human MPNST [9]. All target identifiers from

the external dataset were summarized at the gene level using BioMart [27]. Raw data from all datasets was log2 normalised and genes were ranked according to their mean expression across all samples. Ranked position indicates expression in canine cohort (y-axis) and external human dataset (line extension of the x-axis). Only common genes in canine and human were included in the barcodeplot analysis. Pearson correlation analysis of ranked position was used to confirm significance.

**Cross-species comparison of subtype-specific gene signatures**

To determine which canine transcriptomic cluster most closely corresponded to each of the human MPNST G1 and G2 subtypes, human G1 versus G2 differential expression results and previously generated canine differential expression results between transcriptomic clusters were used for cross-species comparison. Genes significant in both datasets (FDR $\leq 0.05$) were retained and overlap between the human and canine subtype-associated gene sets was evaluated together with the direction of differential expression in each species. The dominant conserved signatures consisted of 193 genes upregulated in human MPNST G1 and canine cluster C2 and 276 genes upregulated in human MPNST G2 and canine cluster C1, indicating that canine cluster C2 most closely corresponded to human MPNST G1, whereas canine cluster C1 most closely corresponded to human MPNST G2. The conserved genes were then assessed by correlation analysis of log fold-change values and visualized in the canine cohort using a row-scaled heatmap generated with the ComplexHeatmap package.

**SingleR**

Raw count data from canine tumors and an extrenal human MPNST dataset [9] were normalized to counts per million (CPM), $\log_2$-transformed, and mean-centered across genes, with low-variance genes filtered out by retaining only those with variance >1. Human MPNST subgroup-specific transcriptional signatures were generated by averaging expression across samples within each subgroup to create a G1/G2 reference signature matrix. Canine samples were annotated using SingleR [11] against the human MPNST signatures, assigning each sample to the closest human methylation subgroup. A heatmap integrating the canine cluster information and SingleR assignments were generated, using the top 100 most variable genes visualized to highlight major transcriptional differences.

**Gene set enrichment analysis**

Canine genes were first mapped to their human orthologs using **Ensembl BioMart.** Genes were filtered for non-missing names and duplicates, retaining the entry with the largest absolute t-statistic per gene to create ranked lists for GSEA. Human Hallmark gene sets (H collection) were obtained from **msigdbr**. GSEA was performed separately for C1 and C2 using the **fgsea** package, with a minimum pathway size of 10 and maximum of 500 genes. Significantly enriched pathways were filtered at an adjusted p-value < 0.05. For visualization, pathway-level enrichment was represented in two complementary ways. First, a **bubble plot** combined pathways from both clusters, with the x-axis showing the gene ratio (number of leading-edge genes divided by total pathway size), the y-axis listing pathways, dot size indicating the number of leading-edge genes, and dot color reflecting the normalized enrichment score (NES). Second, **enrichment plots for selected pathways** were generated, displaying the running enrichment score across the ranked gene list with genes colored according to their differential expression ($\log_2$ fold-change), providing a quantitative and visual summary of pathway-specific enrichment for each cluster.

**Immunohistochemistry**

FFPE tissue sections (2 µm) were mounted on positively charged glass slides (Superfrost™ Plus, Epredia), dried overnight at room temperature, and subsequently incubated at 60°C for 5 minutes. Sections were deparaffinized in xylene and rehydrated through graded ethanol solutions to distilled water. Heat-induced antigen retrieval was performed in a pressure cooker at 98°C for GFAP and Sox10 staining using citrate buffer (Dako). After rinsing in TBS-Tween buffer, slides were processed on an automated staining system, and endogenous peroxidase activity was blocked using a peroxidase blocking solution (Dako). Primary antibody incubation was carried out using anti-GFAP (Dako, Z0334; dilution 1:600), anti-Sox10 (A-2, Santa Cruz, sc-365692; dilution 1:100) and anti-NGFR (Santa Cruz, sc-13577; dilution 1:500). Additional immunohistochemical staining for S100 and Ki-67 was performed on an automated Ventana platform using CC1 (mild) antigen retrieval. Antibodies included S100 (clone 15E2E2, BioGenex, MU058-UL; dilution 1:10,000) and Ki-67 (clone MIB-1, Dako/Agilent, M7240; dilution 1:50). Following washing steps, detection was performed using HRP-based polymer systems, namely the Mach4 HRP system (Biocare) for GFAP and the ZytoChem Plus HRP polymer anti-mouse system for for Sox10 and NGFR, and the signal was visualized using 3,3′-diaminobenzidine (DAB) as chromogen. Slides were counterstained with hematoxylin (Gill II), rinsed, dehydrated, and coverslipped using an automated coverslipping system. Appropriate control tissues were included for each staining run (brain tissue for GFAP, intestinal tissue for NGFR, appendix for S100, and tonsil for Ki-67).

S100 expression was assessed semiquantitatively with regards to staining intensity and proportion of positive tumour cells, whereas Ki-67 was evaluated as a nuclear labelling index in hotspot areas. All stainings were reviewed by an expert human soft tissue pathologist (AJ).

Immunohistochemical staining for phospho-ERK and Histone H3k27me3 was performed on 2 µm FFPE tissue sections following the initial deparaffinization and rehydration protocol described above. Heat-induced antigen retrieval for both markers was achieved using a citrate-based solution (pH 6.0) for 20 minutes. Following endogenous peroxidase blocking and washing steps, sections were incubated with primary antibodies against phospho-ERK (Cell Signaling Technology, 4370; dilution 1:200) and Histone H3k27me3 (Cell Signaling Technology, 9733; dilution 1:200). Detection and signal visualization were carried out using the previously described HRP-based polymer system with 3,3′-diaminobenzidine (DAB) as the chromogen. Slides were subsequently counterstained with hematoxylin, dehydrated, and coverslipped using the automated system. To verify staining validity, appropriate positive control tissues were included in each run (tonsil tissue for phospho-ERK and adenocarcinoma tissue for Histone H3k27me3).

**Graphical display of results**

Previously mentioned R packages were used for visualization of the results, and panels were assembled using Affinity Publisher 2.

**References**


1. Bolger AM, Lohse M, Usadel B. Trimmomatic: A flexible trimmer for Illumina sequence data. Bioinformatics. 2014 Aug 1;30(15):2114–20. doi:10.1093/bioinformatics/btu170 PubMed PMID: 24695404.

2. Bray NL, Pimentel H, Melsted P, Pachter L. Near-optimal probabilistic RNA-seq quantification. Nat Biotechnol. 2016 May 1;34(5):525–7. doi:10.1038/nbt.3519 PubMed PMID: 27043002.
3. Robinson MD, Oshlack A. A scaling normalization method for differential expression analysis of RNA-seq data. Genome Biol. 2010 Mar 2;11(3). doi:10.1186/gb-2010-11-3-r25 PubMed PMID: 20196867.
4. Ritchie ME, Phipson B, Wu D, Hu Y, Law CW, Shi W, Smyth GK. Limma powers differential expression analyses for RNA-sequencing and microarray studies. Nucleic Acids Res. 2015 Jan 6;43(7):e47. doi:10.1093/nar/gkv007 PubMed PMID: 25605792.
5. Wickham H. Springer-Verlag New York. https://ggplot2.tidyverse.org. 2016. ggplot2: Elegant Graphics for Data Analysis. ISBN 978-3-319-24277-4.
6. Gu Z. Complex heatmap visualization. iMeta. 2022 Sep;1(3). doi:10.1002/imt2.43
7. Gu Z, Eils R, Schlesner M. Complex heatmaps reveal patterns and correlations in multidimensional genomic data. Bioinformatics. 2016 Sep 15;32(18):2847–9. doi:10.1093/bioinformatics/btw313
8. Hedan B, Rault M, Abadie J, Ulvé R, Botherel N, Devauchelle P, Copie-Bergman C, Cadieu E, Parrens M, Alten J. PTPN11 mutations in canine and human disseminated histiocytic sarcoma. Int J Cancer. 2020(6). doi:10.1002/ijc.32991ï
9. Suppiah S, Mansouri S, Mamatjan Y, Liu JC, Bhunia MM, Patil V, Rath P, Mehani B, Heir P, Bunda S, Velez-Reyes GL, Singh O, Ijad N, Pirouzmand N, Dalcourt T, Meng Y, Karimi S, Wei Q, Nassiri F, Pugh TJ, Bader GD, Aldape KD, Largaespada DA, Zadeh G. Multiplatform molecular profiling uncovers two subgroups of malignant peripheral nerve sheath tumors with distinct therapeutic vulnerabilities. Nat Commun. 2023 May 10;14(1):2696. doi:10.1038/s41467-023-38432-6
10. Durinck S, Spellman PT, Birney E, Huber W. Mapping identifiers for the integration of genomic datasets with the R/ Bioconductor package biomaRt. Nat Protoc. 2009;4(8):1184–91. doi:10.1038/nprot.2009.97 PubMed PMID: 19617889.
11. Aran D, Looney AP, Liu L, Wu E, Fong V, Hsu A, Chak S, Naikawadi RP, Wolters PJ, Abate AR, Butte AJ, Bhattacharya M. Reference-based analysis of lung single-cell sequencing reveals a transitional profibrotic macrophage. Nat Immunol. 2019 Feb 1;20(2):163–72. doi:10.1038/s41590-018-0276-y PubMed PMID: 30643263.
